# Supplementary material for: COZOID: contact zone identifier for visual analysis of protein-protein interactions
Source: BMC Bioinformatics. 2018 Apr 6;19:125. doi: 10.1186/s12859-018-2113-6 (PMC5889581; doi:10.1186/s12859-018-2113-6)
Supplement: Supplementary file 2 — Software build. Executable binary file of the software tool. (ZIP 118,784 kb) [file 12859_2018_2113_MOESM2_ESM.zip › cozoid/platform/docs/testng-6.5.1-javadoc.zip!/org/testng/xml/XmlClass.html]

XmlClass (TestNG)


---


|  |  |  |
| --- | --- | --- |
| **Overview** \| **Package** \| **Class** \| **Tree** \| **Deprecated** \| **Index** \| **Help** | |  |
| PREV CLASS   **NEXT CLASS** | **FRAMES**    **NO FRAMES**     **All Classes** |  |
| SUMMARY: NESTED \| FIELD \| CONSTR \| METHOD | DETAIL: FIELD \| CONSTR \| METHOD |  |


---


## org.testng.xml Class XmlClass

```
java.lang.Object
  org.testng.xml.XmlClass
```

**All Implemented Interfaces:**: java.io.Serializable, java.lang.Cloneable

---

``` public class XmlClass extends java.lang.Object implements java.io.Serializable, java.lang.Cloneable ```

This class describes the tag  in testng.xml.

**Author:**
:   Cedric Beust

**See Also:**: Serialized Form

---

**Constructor Summary**

`XmlClass()`

`XmlClass(java.lang.Class cls)`

`XmlClass(java.lang.Class cls,
boolean loadClasses)`

`XmlClass(java.lang.String name)`

`XmlClass(java.lang.String name,
boolean loadClasses)`

`XmlClass(java.lang.String className,
int index)`

`XmlClass(java.lang.String className,
int index,
boolean loadClasses)`


| **Method Summary** | |
| --- | --- |
| `java.lang.Object` | `clone()`             Clone an XmlClass by copying all its components. |
| `boolean` | `equals(java.lang.Object obj)` |
| `java.util.List<java.lang.String>` | `getExcludedMethods()` |
| `java.util.List<XmlInclude>` | `getIncludedMethods()` |
| `int` | `getIndex()`             Note that this attribute does not come from the XML file, it's calculated internally and represents the order in which this class was found in its <test> tag. |
| `java.lang.String` | `getName()` |
| `java.lang.Class` | `getSupportClass()` |
| `int` | `hashCode()` |
| `static java.lang.String` | `listToString(java.util.List<java.lang.Integer> invocationNumbers)` |
| `boolean` | `loadClasses()` |
| `void` | `setClass(java.lang.Class className)` |
| `void` | `setExcludedMethods(java.util.List<java.lang.String> excludedMethods)` |
| `void` | `setIncludedMethods(java.util.List<XmlInclude> includedMethods)` |
| `void` | `setIndex(int index)` |
| `void` | `setName(java.lang.String name)` |
| `java.lang.String` | `toString()` |
| `java.lang.String` | `toXml(java.lang.String indent)` |

**Methods inherited from class java.lang.Object**

`finalize, getClass, notify, notifyAll, wait, wait, wait`

**Constructor Detail**

### XmlClass

```
public XmlClass()
```

---


### XmlClass

```
public XmlClass(java.lang.String name)
```

---


### XmlClass

```
public XmlClass(java.lang.String name,
                boolean loadClasses)
```

---


### XmlClass

```
public XmlClass(java.lang.Class cls)
```

---


### XmlClass

```
public XmlClass(java.lang.Class cls,
                boolean loadClasses)
```

---


### XmlClass

```
public XmlClass(java.lang.String className,
                int index)
```

---


### XmlClass

```
public XmlClass(java.lang.String className,
                int index,
                boolean loadClasses)
```


**Method Detail**

### getSupportClass

```
public java.lang.Class getSupportClass()
```

:   **Returns:**: Returns the className.

---


### setClass

```
public void setClass(java.lang.Class className)
```

:   **Parameters:**: `className` - The className to set.

---


### getExcludedMethods

```
public java.util.List<java.lang.String> getExcludedMethods()
```

:   **Returns:**: Returns the excludedMethods.

---


### setExcludedMethods

```
public void setExcludedMethods(java.util.List<java.lang.String> excludedMethods)
```

:   **Parameters:**: `excludedMethods` - The excludedMethods to set.

---


### getIncludedMethods

```
public java.util.List<XmlInclude> getIncludedMethods()
```

:   **Returns:**: Returns the includedMethods.

---


### setIncludedMethods

```
public void setIncludedMethods(java.util.List<XmlInclude> includedMethods)
```

:   **Parameters:**: `includedMethods` - The includedMethods to set.

---


### getName

```
public java.lang.String getName()
```

:   **Returns:**: Returns the name.

---


### setName

```
public void setName(java.lang.String name)
```

:   **Parameters:**: `name` - The name to set.

---


### loadClasses

```
public boolean loadClasses()
```

:   **Returns:**: true if the classes need to be loaded.

---


### toString

```
public java.lang.String toString()
```

:   **Overrides:**: `toString` in class `java.lang.Object`

---


### toXml

```
public java.lang.String toXml(java.lang.String indent)
```

---


### listToString

```
public static java.lang.String listToString(java.util.List<java.lang.Integer> invocationNumbers)
```

---


### clone

```
public java.lang.Object clone()
```

:   Clone an XmlClass by copying all its components.

    :   **Overrides:**: `clone` in class `java.lang.Object`

---


### getIndex

```
public int getIndex()
```

:   Note that this attribute does not come from the XML file, it's calculated
    internally and represents the order in which this class was found in its
    <test> tag. It's used to calculate the ordering of the classes
    when preserve-order is true.

---


### setIndex

```
public void setIndex(int index)
```

---


### hashCode

```
public int hashCode()
```

:   **Overrides:**: `hashCode` in class `java.lang.Object`

---


### equals

```
public boolean equals(java.lang.Object obj)
```

:   **Overrides:**: `equals` in class `java.lang.Object`


---


|  |  |  |
| --- | --- | --- |
| **Overview** \| **Package** \| **Class** \| **Tree** \| **Deprecated** \| **Index** \| **Help** | |  |
| PREV CLASS   **NEXT CLASS** | **FRAMES**    **NO FRAMES**     **All Classes** |  |
| SUMMARY: NESTED \| FIELD \| CONSTR \| METHOD | DETAIL: FIELD \| CONSTR \| METHOD |  |


---
